# Sleep maturation influences cognitive development of preterm toddlers

**DOI:** 10.1038/s41598-021-95495-5

**Published:** 2021-08-05

**Authors:** Akiko Ando, Hidenobu Ohta, Yuko Yoshimura, Machiko Nakagawa, Yoko Asaka, Takayo Nakazawa, Yusuke Mitani, Yoshihisa Oishi, Masato Mizushima, Hiroyuki Adachi, Yosuke Kaneshi, Keita Morioka, Rinshu Shimabukuro, Michio Hirata, Takashi Ikeda, Rika Fukutomi, Kyoko Kobayashi, Miwa Ozawa, Masahiro Takeshima, Atsushi Manabe, Tsutomu Takahashi, Kazuo Mishima, Isao Kusakawa, Hitoshi Yoda, Mitsuru Kikuchi, Kazutoshi Cho

**Affiliations:** 1 Maternity and Perinatal Care Center, Hokkaido University Hospital, N15, W7, Kita-ku, Sapporo, 060-8638 Japan; 2 Department of Neuropsychiatry, Akita University Graduate School of Medicine, Hondo 1-1-1, Akita, Akita, 010-8543 Japan; 3 Department of Sleep-Wake Disorders, National Institute of Mental Health, National Center of Neurology and Psychiatry, 4-1-1 Ogawa-higashi-cho, Kodaira, Tokyo 187-8553 Japan; 4 Department of Psychiatry, Asai Hospital, 38-1 Togane, Chiba, 283-0062 Japan; 5 Department of Pediatrics, St. Luke’s International Hospital, 9-1 Akashi-cho, Chuo-ku, Tokyo, 104-8560 Japan; 6 Pediatric Nursing, Graduate School of Nursing Science, St. Luke’s International University, 10-1 Akashi-cho, Chuo-ku, Tokyo, 104-0044 Japan; 7 Department of Neonatology, Toho University Omori Medical Center, 6-11-1 Omori-nishi, Ota-ku, Tokyo, 143-8541 Japan; 8 Faculty of Health Sciences, Hokkaido University, N12, W5, Kita-ku, Sapporo, 060-0812 Japan; 9 Research Center for Child Mental Development, Kanazawa University, 13-1 Takara-machi, Kanazawa, 920-8640 Japan; 10 Institute of Human and Social Sciences, Kanazawa University, Kakuma-machi, Kanazawa, 921-1192 Japan; 11 Department of Pediatrics, Kanazawa University, 13-1 Takara-machi, Kanazawa, 920-8640 Japan; 12 Department of Pediatrics, Japanese Red Cross Medical Center, 4-1-22 Hiroo, Shibuya-ku, Tokyo, 150-8935 Japan; 13 Department of Neonatology, Sapporo City General Hospital, N11, W13, Chuo-ku, Sapporo, 060-8604 Japan; 14 Department of Pediatrics, Akita University Graduate School of Medicine, Hondo 1-1-1, Akita, Akita 010-8543 Japan; 15 Department of Pediatrics, Hokkaido University Graduate School of Medicine, N15, W7, Kita-ku, Sapporo, 060-8638 Japan

**Keywords:** Paediatric research, Paediatrics

## Abstract

Our recent study on full-term toddlers demonstrated that daytime nap properties affect the distribution ratio between nap and nighttime sleep duration in total sleep time but does not affect the overall total amount of daily sleep time. However, there is still no clear scientific consensus as to whether the ratio between naps and nighttime sleep or just daily total sleep duration itself is more important for healthy child development. In the current study, to gain an answer to this question, we examined the relationship between the sleep properties and the cognitive development of toddlers born prematurely using actigraphy and the Kyoto scale of psychological development (KSPD) test. 101 premature toddlers of approximately 1.5 years of age were recruited for the study. Actigraphy units were attached to their waist with an adjustable elastic belt for 7 consecutive days and a child sleep diary was completed by their parents. In the study, we found no significant correlation between either nap or nighttime sleep duration and cognitive development of the preterm toddlers. In contrast, we found that stable daily wake time was significantly associated with better cognitive development, suggesting that sleep regulation may contribute to the brain maturation of preterm toddlers.

## Introduction

Children’s sleep architecture develops rapidly during the first 5 years of life bringing about dramatic changes in their sleep patterns. During this period, the duration and frequency of daytime naps diminishes and they begin to adopt a more consolidated nighttime sleep, like that in adults. In a previous study, we examined the sleep properties of full-term toddlers approximately 1.5 years of age and demonstrated that nap duration directly influences the distribution ratio between nap and nighttime sleep but does not affect overall total daily sleep duration^1^. There is, however, still an ongoing debate surrounding the two hypotheses on whether either nap or nighttime sleep contributes more to the proper cognitive development of children or whether appropriate intellectual development depends merely on daily total sleep duration^2,3^.

In the current study, to gain an answer to this question, we examined the relationship between the sleep properties and cognitive development of 101 toddlers who had been born prematurely (preterm toddlers) and whose physiological and psychological data had been systematically collected from birth. Focusing on the early developmental stage of approximately 1.5 years of age, when the basic sleep structure of young children has been reported to be established^1,4–14^, we examined the effects of sleep maturation on the cognitive development of the preterm toddlers in order to find which sleep variables, such as nap, nighttime sleep, total sleep duration, or other sleep variables, contribute to their cognitive development.

## Results

### Sleep properties of the preterm toddlers

**Figure 1:**
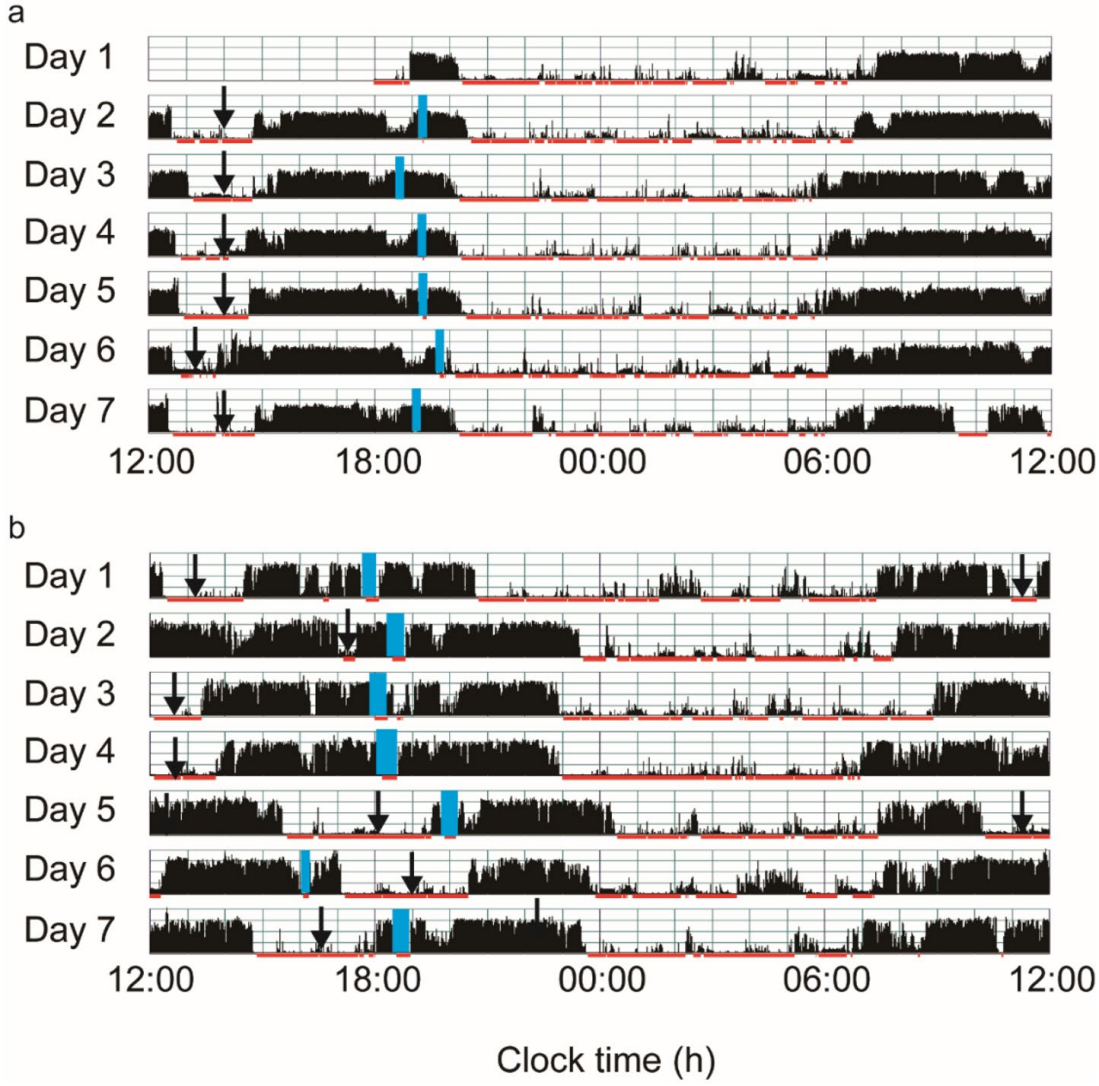
The actograms show representative daily activity-rest patterns of preterm toddlers with stable daily wake times (**a**) (an infant with DQ96) and unstable daily wake times (**b**) (an infant with DQ73). The vertical axis shows the 7 consecutive observation days and the horizontal axis shows the course of each 24 h day from 12:00 h (00:00 pm). Activity counts per minute are represented by the height of the vertical black bars on each actogram. The arrows and the blue rectangles indicate naps and bathing periods, respectively. The red underlines are the periods that were automatically judged as sleep periods by the actigraph software. Note that the wake times are recognized as relatively regular starts of the vertical black bars at around 6:00 h (06:00 am) in (**a**) but as irregular starts of the vertical black bars between 6:00 h (06:00 am) and 9:00 h (09:00 am) in (**b**).

**Figure 2:**
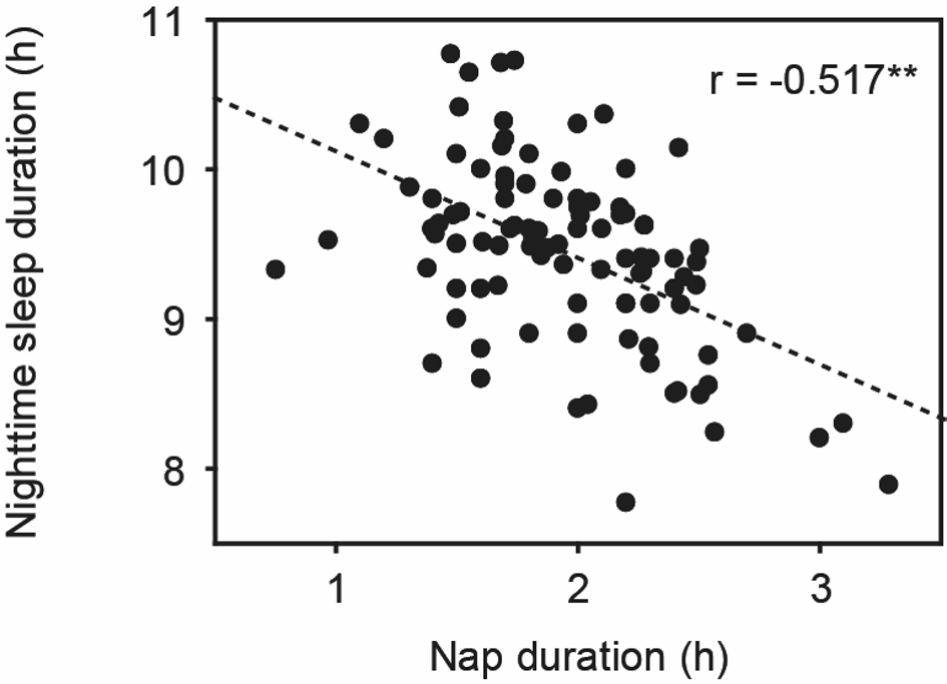
Correlations of nighttime sleep duration with nap duration in preterm toddlers of approximately 1.5 years of age (**p < 0.01).

The characteristics of the 101 toddlers are shown in Table 1. No significant difference in characteristics by gender was detected (p < 0.05). The toddlers’ sleep arrangements are shown in Table 2. No significant difference in sleep arrangements by gender was detected except for “Putting children to sleep with formula” (p = 0.024), suggesting that more male toddlers were fed with formula at onset of nighttime sleep. The toddlers’ sleep variables such as bedtime, wake time, nighttime sleep duration, and nap duration are shown in Table 3 (Supplementary Data 1 and 2). No differences were found between boys and girls among the 15 different sleep variables (t-test, p > 0.05) except for daily variation in wake time, nap onset time and sleep efficiency. Boys were found to have more daily variation in wake time (p = 0.048), lower sleep efficiency (p = 0.035), and an earlier nap onset time (p = 0.017) compared to girls. Figure 1 demonstrates the representative daily activity-rest patterns of the approximately 1.5-year-old toddlers, indicating the existence of various nap patterns among the toddlers. There was a significant negative correlation between nap duration and nighttime sleep duration (r = − 0.517, p = 0.000), suggesting that longer nap duration induces shorter nighttime sleep duration (Fig. 2), as we previously reported^1^.

**Table 1:** Characteristics of participants by gender (median for Apgar score; mean ± s.d. or number for other variables).

	Total (n = 101)	Boys (n = 44)	Girls (n = 57)	p-value
**Gestational age at birth (weeks)**	28.9 ± 2.6	28.9 ± 2.4	28.9 ± 2.8	0.421
	(22.9–35.1)	(24.4–33.9)	(22.9–35.1)	
**Birth weight (g)**	1012 ± 295	1009 ± 317	1014 ± 279	0.105
	(474–1495)	(530–1495)	(474–1488)	
**Extremely low birth weight (< 1000 g)**	49	23	26	0.626
	(48.5%)	(22.8%)	(25.7%)	
**Small for gestational age(< 10th centile)**	48	24	24	0.149
	(47.5%)	(23.8%)	(23.8%)	
**Birth length (cm)**	35.4 ± 3.8	35.5 ± 4.1	35.3 ± 3.7	0.210
	(25.0–44.0)	(27.0–41.1)	(25.0–44.0)	
**Maternal age at birth (years)**	35.6 ± 4.7	35.7 ± 5.2	35.5 ± 4.4	0.210
	(24–48)	(27–48)	(24–44)	
**Birth order**				
First born	68	29	39	0.833
	(67.3%)	(28.7%)	(38.6%)	
Subsequently born	33	15	18	
	(32.7%)	(14.9%)	(17.8%)	
**Apgar score**				
1 min	7	8	6	0.686
	(1–9)	(1–9)	(1–9)	
5 min	9	9	9	0.266
	(2–10)	(3–10)	(2–9)	
**RDS**	73	32	41	0.555
	(72.3%)	(31.7%)	(40.6%)	
**Prolonged ventilation (> 7 days)**	30	12	18	0.403
	(29.7%)	(11.9%)	(17.8%)	
**Non-significant CLD**	44	19	25	0.467
	(43.6%)	(18.8%)	(24.8%)	
**Months of age at actigraph recording**	19.6 ± 1.0	19.5 ± 1.0	19.7 ± 1.2	0.341
	(17.0–22.8)	(17.1–21.8)	(18.1–22.8)	
**DQ scores of KSPD**	93.4 ± 10.8	91.0 ± 11.9	95.3 ± 9.7	0.053
	(66–113)	(66–111)	(71–113)	

*RDS* respiratory distress syndrome, *Non-significant CLD* non-significant chronic lung disease.

**Table 2:** Sleep arrangements and sleep variables by gender (number or mean ± s.d., *p < 0.05).

		Boys (n = 44)	Girls (n = 57)	p-value
**Home environment**				
Siblings				
Yes		29 (28.7%)	39 (38.6%)	
No		15 (14.9%)	18 (17.8%)	0.790
Child having own room				
Yes		2 (2.0%)	2 (2.0%)	
No		42 (41.6%)	55 (54.5%)	0.791
Co-sleeping with parents				
Yes		33 (32.7%)	45 (44.6%)	
No		11 (10.9%)	12 (11.9%)	0.639
**Nighttime feeding**				
Breastmilk		16 (15.8%)	15 (14.9%)	
Formula		6 (5.9%)	9 (8.9%)	
No feeding		22 (21.8%)	33 (32.7%)	0.555
**Putting children to sleep with formula**				
Yes		22 (21.8%)	16 (15.8%)	
No		22 (21.8%)	41 (40.6%)	0.024*
**Nap during daytime**				
Yes		44 (43.6%)	57 (56.4%)	
No		0 (0.0%)	0 (0.0%)	n.a
**Child attending kindergarten**				
Yes		14 (13.9%)	17 (16.8%)	
No		30 (29.7%)	40 (39.6%)	0.829
**Bed time**				
Weekday	20:56 ± 0:41	20:44 ± 0:39	21:06 ± 0:41	0.701
Weekend	20:58 ± 0:50	20:50 ± 0:45	21:03 ± 0:53	0.254
p-value	0.122	0.513	0.097	
**Wake time**				
Weekday	06:56 ± 0:39	06:56 ± 0:37	06:57 ± 0:40	0.534
Weekend	07:04 ± 0:48	07:01 ± 0:49	07:07 ± 0:48	0.987
p-value	0.110	0.166	0.300

**Table 3:** Sleep variables by gender (mean ± s.d., *p < 0.05).

	Total (n = 101)	Boys (n = 44)	Girls (n = 57)	p-value
**Nighttime sleep variables**				
Bed time	20:58 ± 0:42	20:46 ± 0:38	21:07 ± 0:42	0.436
Sleep onset time	21:28 ± 0:43	21:18 ± 0:39	21:35 ± 0:45	0.200
Wake time	6:58 ± 0:40	6:57 ± 0:39	7:00 ± 0:41	0.532
Daily variation in sleep onset time	33.5 ± 18.7	35.4 ± 19.8	32.1 ± 17.9	0.741
Daily variation in wake time	33.0 ± 15.7	35.1 ± 17.9	31.3 ± 13.7	0.048*
Sleep latency (min)	29.7 ± 14.7	33.0 ± 15.7	27.1 ± 13.3	0.317
Nighttime sleep duration (h)	9.4 ± 0.6	9.5 ± 0.6	9.4 ± 0.6	0.842
Total sleep duration (h)	11.3 ± 0.6	11.4 ± 0.6	11.3 ± 0.6	0.608
Sleep efficiency (%)	85.7 ± 9.0	83.7 ± 11.0	87.2 ± 6.9	0.035*
WASO (wake after sleep onset)(min)	82.6 ± 51.8	94.0 ± 61.4	73.8 ± 41.4	0.056
Nighttime activity (counts/min)	26.4 ± 10.2	29.0 ± 12.3	24.3 ± 7.8	0.111
**Daytime sleep variables**				
Daytime activity (counts/min)	239 ± 19.4	240 ± 22.8	239 ± 16.6	0.183
Nap duration (h)	2.0 ± 0.4	2.0 ± 0.4	1.9 ± 0.5	0.068
Nap onset time	12:37 ± 1:04	12:23 ± 0:48	12:48 ± 1:12	0.017*
Nap end time	15:00 ± 0:52	14:50 ± 0:42	15:08 ± 0:57	0.086

### Effects of sleep on cognitive development of the preterm toddlers

Before logistic regression analysis, univariate regression analysis was performed in order to select variables (Table 4). Next, to evaluate possible factors contributing to the toddlers’ cognitive development, we performed a logistic regression analysis for the effects of birth profiles, respiratory complications, sleep variables and sleep arrangements on the cognitive development (DQ (Developmental Quotient) scores of the KSPD test) of the preterm toddlers (Table 5). According to analysis of DQ scores in model 1, which was adjusted for birth profile characteristics such as gender and birth weight, no significant odds ratios (ORs) for toddlers with a DQ score of ≥ 93.4 (mean) were found. In model 2, which adds the respiratory complications of prolonged ventilation and non-significant chronic lung disease (CLD) to model 1, no significant ORs for toddlers with a DQ score of ≥ 93.4 (mean) were found. In model 3, which adds the sleep variables of daily variation (standard deviation) of wake time, daily variation (standard deviation) of sleep onset time, sleep onset time, and total sleep duration to model 2, the ORs for toddlers with a DQ score of ≥ 93.4 (mean) were 0.964 (p = 0.014) for daily variation of wake time, indicating that greater daily variation of wake time is a significant predictor of lower DQ in toddlers, but failed to find any significant correlations with other sleep variables. In model 4, which adds the sleep arrangement factors of co-sleeping with parents, child attending kindergarten, and nighttime formula feeding to model 3, the ORs for toddlers with a DQ score of ≥ 93.4 (mean) were also 0.964 (p = 0.014) for daily variation of wake time, again indicating that greater daily variation of wake time is a significant predictor of lower DQ in toddlers, but failed to find any significant correlations with other variables.

**Table 4:** The associations of birth profiles, respiratory complications, sleep variables, and sleep arrangements and DQ scores evaluated by univariate regression analysis (**p < 0.01, *p < 0.05).

DQ-related factors	r	R^2^	p-value
**Birth profiles**			
Gender	− 0.197	0.039	0.048*
Birth weight	0.169	0.028	0.092
Gestational age	0.160	0.025	0.111
Maternal age at birth	0.073	0.005	0.471
Birth order	0.044	0.002	0.664
**Respiratory complications**			
Prolonged ventilation (> 7 days )	− 0.198	0.039	0.048*
Non-significant CLD	− 0.195	0.038	0.050
RDS	− 0.073	0.005	0.468
**Sleep variables**			
Daily variation in wake time	− 0.346	0.120	0.000**
Daily variation in sleep onset time	− 0.279	0.078	0.005**
Total sleep duration	0.205	0.042	0.040*
Sleep onset time	− 0.189	0.036	0.058
Bed time	− 0.143	0.021	0.152
Nighttime sleep duration	0.125	0.016	0.213
Nap duration	0.088	0.008	0.380
Wake time	− 0.076	0.006	0.449
Total bed duration	0.067	0.005	0.502
Sleep efficiency	0.061	0.004	0.543
Sleep latency	− 0.040	0.000	0.693
Night wakings	0.031	0.001	0.762
WASO	− 0.015	0.000	0.881
Nap end time	− 0.011	0.000	0.912
Nap onset time	0.008	0.000	0.934
**Sleep arrangements**			
Co-sleeping with parents	0.229	0.052	0.021*
Nighttime formula feeding	− 0.812	0.033	0.069
Child attending kindergarten	0.145	0.021	0.148
Putting children to sleep with formula	− 0.109	0.012	0.277
Nighttime breast feeding	0.074	0.015	0.460
No feeding during nighttime	0.061	0.004	0.545
Child having own room	− 0.060	0.004	0.553

*RDS* respiratory distress syndrome, *Non-significant CLD* non-significant chronic lung disease.

**Table 5:** Logistic regression analysis of DQ scores of toddlers with birth profiles, respiratory complications, sleep variables, and sleep arrangements (OR, 95% CI, **p < 0.01,* p < 0.05).

Variables	Model 1, OR (CI)	Model 2, OR (CI)	Model 3, OR (CI)	Model 4, OR (CI)
Gender	N.S	N.S	N.S	N.S
Birth weight	N.S	N.S	N.S	N.S
Prolonged ventilation (> 7 days)		N.S	N.S	N.S
Non-significant CLD		N.S	N.S	N.S
Daily variation of wake time (h)	–	–	0.964 (0.935, 0.993)*	0.964 (0.935,0.993)*
Daily variation of sleep onset time (h)	–	–	N.S	N.S
Sleep onset time	–	–	N.S	N.S
Total sleep duration	–	–	N.S	N.S
Co-sleeping with parents	–	–	–	N.S
Child attending kindergarten	–	–	–	N.S
Nighttime formula feeding	–	–	–	N.S
p-value	N.S	N.S	0.008**	0.008**
R^2^ (Cox-Snell)	N.S	N.S	0.068	0.068

*RDS* respiratory distress syndrome, *Non-significant CLD* non-significant chronic lung disease, *N.S.* not significant.

## Discussion

The present study indicates three significant findings concerning the sleep properties of preterm toddlers at approximately 1.5 years of age. First, our study describes a new finding that only daily variation of wake time, a sleep regulatory variable, is significantly associated with the cognitive development (the DQ scores of the KSPD test) of preterm toddlers in the logistic regression analysis (Table 5). This is inconsistent with a current working hypothesis that the DQ scores of preterm toddlers are significantly influenced by nap and/or nighttime sleep duration^2,3^. Rather, the maturation of the sleep regulatory mechanism, which controls wake time, contributes to or reflects the levels of preterm toddlers’ cognitive development. This is partly supported by the findings of previous studies in which the daily variation of wake time of full-term infants has been reported to decrease as infants mature^15^. Present data also suggests that, among toddlers, the cortex, which is responsible for cognitive functions, may also play an important role in sleep/wake transition as the final destination of the output from the GABAergic and/or the orexinergic pathway. It has been known that the sleep/wake transition of animals is modulated by their cognitive status, which is affected by environmental conditions such as feeding, mating, and predation. In particular, the orexinergic neurons of the lateral hypothalamus (LH) have been reported to increase wake in response to stress such as from reduced food availability^16^. So far, however, rather than the cortex, the GABAergic neurons of the ventrolateral preoptic area (VLPO) and brainstem and/or the LH orexinergic neurons have been hypothesized to mainly control the transition between wake and sleep status in mammals^16^.

The second significant finding is that the current study with preterm toddlers also agrees with a finding from our previous study with full-term toddlers, namely that there is a significant negative correlation between nap duration and nighttime sleep duration^1^, suggesting that longer nap durations may also lead to shorter nighttime sleep durations in preterm toddlers (Fig. 2). This indicates that the balance between nap and nighttime sleep duration is a strong sleep regulatory mechanism and also that we may be able to control the nighttime sleep duration of preterm and term toddlers effectively by controlling their nap durations. This is quite different from adults’ sleep regulatory system in which the circadian sleep mechanism plays a more powerful role, resulting in that adults do not have naps but only nighttime sleep^12^.

The third significant finding is that sex-based differences existed among toddlers in their cognitive development and sleep variables (Table 1 & 3). In cognitive development, the female toddlers had higher DQ scores than the male toddlers. This is consistent with the results of previous studies, in which increased intraventricular hemorrhage (IVH) and prolonged ventilatory support from pulmonary diseases among male preterm infants was reported to have contributed to their reduced cognitive development^17,18^. A group from Karolinska University Hospital speculates that IVH and prolonged ventilatory support may enlarge the sexual brain dimorphism already existing at the early developmental stage, leading to delayed myelination and lower white matter volumes in male brains, which may result in lower cognitive functions in preterm male toddlers^17^. In sleep variables, female toddlers had significantly less daily variation in wake time, higher sleep efficiency and later nap onset time, which may reflect more mature sleep regulatory mechanisms being associated with toddlers’ cortical function as we previously discussed.

Several concerns warrant consideration in the present study. First, this study did not examine whether the cortical maturation of toddlers’ brains may affect either their sleep regulatory mechanism and/or cognitive functions. That is, there is a possibility that unstable wake time may simply reflect toddlers’ brain immaturity. To investigate this possibility, we would have to artificially improve or hamper toddlers’ intellectual development and evaluate its effects on sleep regulation. However, such an experimental design has not been scientifically established nor, even if it were, could be ethically approved for use in human studies. Second, although the sleep habits of toddlers are affected by those of their parents, especially their mothers^19^, the present study did not investigate the sleep habits of the parents themselves. Third, several sleep variables related to birth profiles, such as gestational age at birth, were not added as a dependent variable to the logistic regression analysis of the DQ scores of the toddlers to avoid multicollinearity between birth weight and gestational age at birth, although previous studies indicated significant association between gestational age at birth and brain development using psychological assessments and physiological measurements such as those made by EEG^20–23^ (Table 5 and Supplementary Data 3–5). Fourth, although the sleep habits of toddlers would also be affected by their temperament such as mood, adaptability to a new situation, attention span or sensory threshold to stimuli or pain, the present study did not investigate the effect of toddlers’ temperament on sleep variables^21^. Fifth, although co-sleeping with parents would have similar positive effects to those of kangaroo care on the cognitive development of preterm toddlers^24^, the present study was not able to investigate possible significant effect of co-sleeping on toddlers’ DQs as we could not be sure if the preterm toddlers had co-slept with their parents continuously since their discharge from NICUs, or whether they had begun to sleep separately before reaching one year of age in compliance with SIDS prevention recommendations^25^.

## Methods

### Participants

Preterm toddlers of approximately 1.5 years of age were recruited from Hokkaido University Hospital (Sapporo, Japan), Sapporo City Hospital (Sapporo, Japan), St. Luke’s International Hospital (Tokyo, Japan), Toho University Hospital (Tokyo, Japan), Japanese Red Cross Medical Center (Tokyo, Japan) and Kanazawa University Hospital (Kanazawa, Japan). Inclusion criteria were as follows: (1) preterm birth [defined as being born at less than 36 weeks’ gestational age and having a birth weight of less than 1500 g (very low birth weight)] and (2) the absence of chromosomal or other major genetic abnormalities, suspected neuromuscular disorders, intraventricular hemorrhage or significant chronic lung disease (CLD). Non-significant CLD was not considered a factor for exclusion. We defined non-significant CLD as requiring ventilation or/and oxygen at 36 weeks corrected gestational age but not at discharge. Exclusion criteria was parental language difficulties. Age correction was performed as follows: the duration between expected birth date and actual birth date was subtracted from the actual age to calculate the chronological developmental stage. Of 105 eligible toddlers, 4 were excluded because sleep data were invalid due to technical problems with the activity recording devices or incomplete descriptions in sleep diary. The final sample thus consisted of 101 preterm toddlers (44 boys, 57 girls). The ethics committees of Hokkaido University Hospital, Sapporo City Hospital, St. Luke’s International Hospital, Toho University Hospital, Japanese Red Cross Medical Center, Kanazawa University Hospital and Akita University Hospital approved the study protocol (UMIN000021153) and all procedures were carried out in accordance with the approved guidelines. Written informed consent was obtained from the parents.

### Activity and sleep assessment

For activity and sleep assessment we used actigraphy and sleep diaries, as previously described^1^. Briefly, the parents were instructed to attach Actigraphs (Micro-mini RC, Ambulatory Monitoring Inc., NY, USA) to their child’s waist with an adjustable elastic belt for 7 consecutive days^1^. The activity data recorded by the Actigraph were later downloaded using ActMe software (ver. 3.10.0.3, Ambulatory Monitoring Inc., NY, USA), and then sleep measurements were analyzed using Action-W software (ver. 2.4.20, Ambulatory Monitoring Inc., NY, USA). Time intervals during the study when the Actigraph was removed, for example, during bathing, were recorded by parents in a sleep diary^1^. The sleep diary was composed of seven 24-h single-sheet schedules, on which parents were asked to record details such as time of nap, going in/out of bed, bathing and night wakings of which they were aware. Sleep diary data were used to define the scoring interval for actigraphic sleep measurement, according to the procedure outlined by Acebo and colleagues^4^.

### Neurodevelopmental assessment

The assessment of the cognitive function of the preterm infants was performed at approximately 1.5 years of age using the Kyoto Scale of Psychological Development (KSPD) test, as previously described^26^. Briefly, experienced testers who were certified psychologists administered the KSPD test, blinded to the perinatal details of the toddlers. It usually takes approximately 20–40 min to administer. The KSPD is standardized for all subjects ranging from neonates to adults of 29 years of age. This scale consists of 328 items covering the Cognitive-Adaptive area (C-A), Language-Social area (L-S), and Postural-Motor area (P-M). The C-A section assesses non-verbal reasoning and visuospatial perception. The L-S section assesses interpersonal relationships, socialization and verbal abilities. The P-M section assesses fine motor functions. The developmental age is estimated according to the sum score of the three sections. The DQ is then calculated by dividing the developmental age by the chronological age and then multiplying it by 100. A DQ score of 100.6 ± 13.4 represents the mean ± 1 s.d. at the time of standardization^26^.

### Statistical analysis

A Student’s t-test for continuous data or a χ^2^ test for categorical data was performed to compare the characteristics of participants by gender and a χ^2^ test was used to compare the sleep arrangements and sleep variables by gender (Table1, 2, and 3) after confirming that all data fulfilled the requirements for normality and equal variances. Univariate regression analysis was performed before logistic regression analysis (Table 4). The degrees of correlation between the cognitive development parameter (DQ scores of the KSPD test) and birth profiles, respiratory complications, sleep variables, and sleep arrangement factors were assessed using the Spearman correlation test. Only variables with relatively significant values (p < 0.2) in the Spearman correlation tests were included in logistic regression analysis. Logistic regression was used to calculate odds ratios (ORs) with 95% confidence intervals as estimates of effects, with the DQ scores of the toddlers as the outcome variable (Table 5). Statistical analyses were performed with SPSS Statistics 25.0 (IBM Corp. Armonk, NY, USA).

## Supplementary Information

Supplementary Information.
