# Supplementary material for: Sleep maturation influences cognitive development of preterm toddlers
Source: Sci Rep. 2021 Aug 5;11:15921. doi: 10.1038/s41598-021-95495-5 (PMC8342419; doi:10.1038/s41598-021-95495-5)
Supplement: Supplementary file 1 — Supplementary Information. [file 41598_2021_95495_MOESM1_ESM.docx]

**Supplementary information**

Sleep maturation influences cognitive development of preterm toddlers

**Authors:**

Akiko Ando^1^, Hidenobu Ohta^2,3,4^*, Yuko Yoshimura^9,10^, Machiko Nakagawa^5,6,7^, Yoko Asaka^8^*, Takayo Nakazawa^1^, Yusuke Mitani^11^, Yoshihisa Oishi^12^, Masato Mizushima^13^, Hiroyuki Adachi^14^, Yosuke Kaneshi^1^, Keita Morioka^1^, Rinshu Shimabukuro^5^, Michio Hirata^5^, Takashi Ikeda^9^, Rika Fukutomi^6^, Kyoko Kobayashi^6^, Miwa Ozawa^5^, Masahiro Takeshima^2^, Atsushi Manabe^15^, Tsutomu Takahashi^14^, Kazuo Mishima^2^, Isao Kusakawa^5,6^, Hitoshi Yoda^7^, Mitsuru Kikuchi^9^, Kazutoshi Cho^1^

^1^ Maternity and Perinatal Care Center, Hokkaido University Hospital, N15, W7, Kita-ku, Sapporo 060-8638, Japan

^2^ Department of Neuropsychiatry, Akita University Graduate School of Medicine, Hondo 1-1-1, Akita, Akita 010-8543, Japan

^3^ Department of Sleep-Wake Disorders, National Institute of Mental Health, National Center of Neurology and Psychiatry, 4-1-1 Ogawa-higashi-cho, Kodaira, Tokyo 187-8553, Japan

^4^ Department of Psychiatry, Asai Hospital, 38-1 Togane, Chiba 283-0062, Japan

^5^ Department of Pediatrics, St. Luke’s International Hospital, 9-1 Akashi-cho, Chuo-ku, Tokyo 104-8560, Japan

^6^ Pediatric Nursing, Graduate School of Nursing Science, St. Luke's International University, 10-1 Akashi-cho, Chuo-ku, Tokyo 104-0044, Japan

^7^ Department of Neonatology, Toho University Omori Medical Center, 6-11-1 Omori-nishi, Ota-ku, Tokyo 143-8541, Japan

^8^ Faculty of Health Sciences, Hokkaido University, N12, W5, Kita-ku, Sapporo 060-0812, Japan

^9^ Research Center for Child Mental Development, Kanazawa University, 13-1 Takara-machi, Kanazawa 920-8640, Japan

^10^ Institute of Human and Social Sciences, Kanazawa University, Kakuma-machi, Kanazawa 921-1192, Japan

^11^ Department of Pediatrics, Kanazawa University, 13-1 Takara-machi, Kanazawa 920-8640, Japan

^12^ Department of Pediatrics, Japanese Red Cross Medical Center, 4-1-22 Hiroo, Shibuya-ku, Tokyo 150-8935, Japan

^13^ Department of Neonatology, Sapporo City General Hospital, N11, W13, Chuo-ku, Sapporo 060-8604, Japan

^14^ Department of Pediatrics, Akita University Graduate School of Medicine, Hondo 1-1-1, Akita, Akita 010-8543, Japan

^15^ Department of Pediatrics, Hokkaido University Graduate School of Medicine, N15, W7, Kita-ku, Sapporo 060-8638, Japan

**Supplementary Notes**

**Supplementary Data 1. Definition of the terms for sleep variables and sleep arrangement**.

Bed time: The time that the toddler went to bed as defined by sleep diary.

Sleep onset time: The time of the start of nighttime sleep as defined by actigraph. Based on the number of activities, sleep-awake judgment is performed using sleep -awake algorithm of actigraph.

Wake time: The time of the end of nighttime sleep as defined by actigraph.

Daily variation in sleep onset time: Standard deviation of the nighttime sleep onset time during the seven consecutive days of the study period.

Daily variation in wake time: Standard deviation of the wake time after nighttime sleep during the seven consecutive days of the study period.

Sleep latency: The duration of time from bed time to the sleep onset time.

Sleep efficiency: The proportion of actual sleep during bed- in-time (%).

Nighttime sleep duration: Total hour defined as sleep between sleep onset time and wake time.

Total sleep duration: The sum of nighttime sleep and nap duration.

WASO: The sum of wake minutes between sleep onset time and wake time

Night wakings: Number of blocks of continuous wake epochs lasting more than 5 minutes each.

Nap onset time: The time of the start of the first nap of the day as defined by sleep diary.

Nap end time: The time of the end of the last nap of the day as defined by sleep diary

Co-sleeping with parents: Sleeping with parents in the same bed.

**Supplementary Data 2. Sleep variables and nighttime feeding (mean ± s.d. **p<0.01, *p<0.05).**

|  | **Breastmilk (n=31)** | **Formula (n=15)** | **No feeding (n=55)** | **p-value** |
| --- | --- | --- | --- | --- |
| **Nighttime Sleep Variables** |  |  |  |  |
| **Bed time** | **20:59 ± 0:39** | **21:13 ± 0:45** | **20:53 ± 0:42** | **0.262** |
| **Sleep onset time** | **21:32 ± 0:35** | **21:45 ± 0:47** | **21:21± 0:46** | **0.129** |
| **Wake time** | **7:06 ± 0:30** | **7:12 ± 0:36** | **6:50 ± 0:44** | **0.080** |
| **Daily variation in sleep onset time** | **36.5± 19.3** | **41.4 ± 20.8** | **29.7 ± 17.1** | **0.057** |
| **Daily variation in wake time** | **34.6± 17.9** | **33.3 ± 10.8** | **31.9 ± 15.7** | **0.743** |
| **Sleep latency (min)** | **33.0 ± 17.0** | **31.7 ± 15.3** | **27.2 ± 12.7** | **0.184** |
| **Nighttime sleep duration (h)** | **9.4 ± 0.6** | **9.4± 0.8** | **9.4± 0.6** | **0.969** |
| **Total sleep duration (h)** | **11.3 ± 0.6** | **11.4 ± 0.6** | **11.3 ± 0.6** | **0.879** |
| **Sleep efficiency (%)** | **81.6 ± 11.6** | **86.9 ± 5.2** | **87.6 ± 7.5** | **0.010*** |
| **Night wakings** | **14.0± 3.9** | **15.3 ± 5.1** | **13.5 ± 5.4** | **0.459** |
| **WASO (wake after sleep onset)(min)** | **110.3 ± 65.9** | **74.8 ± 29.3** | **69.1 ± 41.1** | **0.001**** |
| **Nighttime activity (counts / min)** | **30.5 ± 14.0** | **26.7 ± 8.0** | **24.0 ± 7.1** | **0.015*** |
| **Daytime Sleep variables** |  |  |  |  |
| **Daytime activity (counts / min)** | **243.1 ±17.0** | **238.6 ± 18.1** | **237.4 ± 21.0** | **0.421** |
| **Nap duration (h)** | **1.9 ± 0.4** | **2.0 ± 0.5** | **2.0 ± 0.5** | **0.748** |
| **Nap onset time** | **12:29 ± 1:08** | **12:42 ± 1:18** | **12:40 ± 0:58** | **0.724** |
| **Nap end time** | **14:51 ± 0:52** | **15:15 ± 1:03** | **15:02 ± 0:49** | **0.350** |

**Supplementary Data 3. Logistic regression analysis of DQ scores of toddlers with birth profiles, respiratory complications, sleep variables, and sleep arrangements (OR, 95%C.I., **p<0.01, *p<0.05).**

| **Variables** | **Model 1, OR (C.I.)** | **Model 2, OR (C.I.)** | **Model 3, OR(C.I.)** | **Model 4, OR(C.I.)** |
| --- | --- | --- | --- | --- |
| **Gender** | **N.S.** | **N.S.** | **N.S.** | **N.S** |
| **Gestational age at birth** | **N.S.** | **N.S.** | **N.S.** | **N.S** |
| **Prolonged ventilation**  **( > 7 days )** |  | **N.S** | **N.S.** | **N.S.** |
| **Non-significant CLD** |  | **N.S** | **N.S.** | **N.S.** |
| **Daily variation of wake time (h)** | - | - | **0.964**  **(0.935, 0.993)*** | **0.964**  **(0.935,0.993)*** |
| **Daily variation of sleep onset time (h)** | **-** | **-** | **N.S.** | **N.S** |
| **Sleep onset time** | **-** | **-** | **N.S.** | **N.S** |
| **Total sleep duration** | **-** | - | **N.S.** | **N.S** |
| **Co-sleeping with parents** | **-** | **-** | **-** | **N.S** |
| **Child attending kindergarten** | **-** | **-** | **-** | **N.S** |
| **Nighttime formula feeding** | **-** | **-** | **-** | **N.S** |
| **p-value** | **N.S.** | **N.S** | **0.008**** | **0.008**** |
| **R^2^ (Cox-Snell)** | **N.S.** | **N.S** | **0.068** | **0.068** |

**Supplementary Data 4.** **The associations of birth profiles, respiratory complications, sleep variables, and sleep arrangements and gestational age at birth evaluated by univariate regression analysis (**p<0.01, *p<0.05).**

| **Gestational age at birth -related factors** | **r** | **R^2^** | **p-value** |
| --- | --- | --- | --- |
| **Birth profiles** |  | | |
| **Birth weight** | **0.750** | **0.562** | **0.000**** |
| **Maternal age at birth** | **0.159** | **0.025** | **0.112** |
| **Birth order** | **-0.064** | **0.004** | **0.523** |
| **Gender** | **-0.017** | **0.000** | **0.865** |
| **Respiratory complications** |  |  |  |
| **RDS** | **-0.592** | **0.351** | **0.000**** |
| **Prolonged ventilation ( > 7days )** | **-0.611** | **0.373** | **0.000**** |
| **Non-significant CLD** | **-0.500** | **0.351** | **0.000**** |
| **Sleep variables** |  | | |
| **Sleep efficiency** | **0.291** | **0.073** | **0.006**** |
| **WASO** | **-0.268** | **0.072** | **0.007**** |
| **Night wakings** | **-0.239** | **0.057** | **0.016*** |
| **Sleep latency** | **-0.137** | **0.019** | **0.172** |
| **Nap onset time** | **-0.062** | **0.004** | **0.538** |
| **Total bed duration** | **-0.048** | **0.002** | **0.631** |
| **Sleep onset time** | **-0.470** | **0.002** | **0.639** |
| **Daily variation in wake time** | **0.044** | **0.002** | **0.661** |
| **Daily variation in sleep onset time** | **-0.028** | **0.001** | **0.777** |
| **Bed time** | **0.027** | **0.000** | **0.792** |
| **Wake time** | **0.027** | **0.001** | **0.792** |
| **Nap duration** | **-0.014** | **0.000** | **0.887** |
| **Nighttime sleep duration** | **0.014** | **0.000** | **0.891** |
| **Nap end time** | **-0.018** | **0.000** | **0.862** |
| **Total sleep duration** | **0.001** | **0.000** | **0.994** |
| **Sleep arrangements** |  | | |
| **Child having own room** | **0.122** | **0.015** | **0.226** |
| **Child attending kindergarten** | **0.078** | **0.005** | **0.439** |
| **Nighttime feeding** | **-0.025** | **0.001** | **0.805** |
| **Co-sleeping with parents** | **0.025** | **0.001** | **0.815** |

**Supplementary Data 5. Logistic regression analysis of Gestational age at birth with birth profiles, respiratory complications, sleep variables, and sleep arrangements (OR, 95%C.I., **p<0.01, *p<0.05).**

| **Variables** | **Model 1, OR (C.I.)** | **Model 2, OR (C.I.)** | **Model 3, OR (C.I.)** |
| --- | --- | --- | --- |
| **Birth weight** | **1.006 (1.004,1.010)**** | **1.007 (1.004,1.010)**** | **1.007 (1.004,1.010)**** |
| **RDS** | **-** | **0.058 (0.013,0.261)**** | **0.058 (0.013,0.261)**** |
| **Non-significant CLD** | **-** | **N.S** | **N.S** |
| **Prolonged ventilation**  **( > 7 days)** | **-** | **N.S.** | **N.S.** |
| **Sleep efficiency** | **-** | **-** | **N.S.** |
| **p-value** | **0.000**** | **0.000**** | **0.000**** |
| **R^2^ (Cox-Snell)** | **0.352** | **0.465** | **0.465** |

RDS: respiratory distress syndrome

Non-significant CLD: non-significant chronic lung disease
